# Substrate Vibrations during Courtship in Three *Drosophila* species

**DOI:** 10.1371/journal.pone.0080708

**Published:** 2013-11-15

**Authors:** Valerio Mazzoni, Gianfranco Anfora, Meta Virant-Doberlet

**Affiliations:** 1 Research and Innovation Centre, Fondazione Edmund, Mach, San Michele all’Adige, Italy; 2 Department of Entomology, National Institute of Biology, Ljubljana, Slovenia; University of Melbourne, Australia

## Abstract

While a plethora of studies have focused on the role of visual, chemical and near-field airborne signals in courtship of *Drosophila* fruit flies, the existence of substrate-borne vibrational signals has been almost completely overlooked. Here we describe substrate vibrations generated during courtship in three species of the *D. melanogaster* group, from the allegedly mute species *D. suzukii*, its sister species *D. biarmipe*s, and from *D. melanogaster*. In all species, we recorded several types of substrate vibrations which were generated by locomotion, abdominal vibrations and most likely through the activity of thoracic wing muscles. In *D. melanogaster* and *D. suzukii*, all substrate vibrations described in intact males were also recorded in males with amputated wings. Evidence suggests that vibrational signalling may be widespread among *Drosophila* species, and fruit flies may provide an ideal model to study various aspects of this widespread form of animal communication.

## Introduction

Courtship behaviour in *Drosophila* is likely among the most thoroughly investigated mating behaviours (reviewed in 1-5). In courtship, *Drosophila* flies rely on visual [2], auditory [6] and chemosensory [7,8] modalities. Since the first description of *D. melanogaster* sounds in the 1960s [9], a plethora of studies has focused on acoustic communication (reviewed in 2,10,11). Males of most *Drosophila* species produce species-specific courtship songs by vibrating their wings, and females detect near-field air-borne sounds by the Johnston’s organ in their antennae [3,4,12]. In most species, males emit more than one type of courtship song [10,11], and the two most commonly recorded types were termed sine and pulse song [13]. Sine song is a continuous humming sound with a fundamental frequency between 100-350 Hz, while pulse song is composed of trains of pulses with a species-specific interpulse interval [6,11]. Hawaiian picture-wing fruit flies produce complex courtship songs [14], and some species also emit high frequency sounds [15,16]. Courtship songs increase female receptivity, and interpulse interval is an important parameter in species recognition [5,6,11]. The relative importance of different signal modalities differs among species [2,17,18], and in some *Drosophila* species acoustic signals appear not to be involved in courtship behaviour [11,18,19].

Small insects using acoustic communication overcome the scaling problems of available muscle power by using either low frequency air-borne sounds effective only at a range of few mm, or low frequency substrate-borne sounds which carry to larger distances [20]. While in drosophilid fruit flies near-field air-borne sound communication has been extensively studied [4,6,10,11], a potential role of substrate-borne signals in their courtship has mostly been overlooked. Vibrational communication is prevalent in insects [21,22]; however, until recently the substrate-borne signals have been associated primarily with Hawaiian species *D. silvestris* [23-25]. Although a potential role of vibrational signals in *Drosophila* courtship has been implied also in other species [25-29], only recently have substrate-borne signals produced during courtship been recorded in several *Drosophila* species [30].

The spotted wing *D. suzukii* from the *D. melanogaster* group [31-33] is becoming an increasingly important species since it is regarded as an invasive, economically important fruit pest [30]. This is one of the allegedly mute *Drosophila* species, in which courtship is supposed to be based on visual signals [11,34,35]. However, in the *suzukii* subgroup, air-borne sounds have been described in *D. biarmipes* and *D. pulchrella* [36,37]. Furthermore, it has been reported that in this subgroup, males of *D. rajasekari* [38], *D. pulchrella* [37] and *D. biarmipes* [30,37] vibrate the abdomen in dorso-ventral direction. Since in many insects, including Diptera, vibrations of the body are associated with substrate-borne signals [21,30,39,40], we recorded substrate vibrations generated during courtship of *D. suzukii*, its sister species *D. biarmipes*, and in *D. melanogaster*. Vibrations of the body are transmitted to the substrate via the legs [21,41], however, air-borne sounds emitted by insects also induce vibrations in the substrate [42,43]. To determine whether in *Drosophila* wings are involved in production of substrate vibrations, we also recorded vibrations emitted by males from which wings had been removed. 

Our recordings of substrate vibrations revealed previously undescribed acoustic cues in *D. suzukii* and *D. biarmipes*. Our results show that repertoire, as well as temporal and spectral parameters of recorded substrate vibrations differs among species. We describe vibrational components of previously recorded air-borne sounds in *D. biarmipes* and *D. melanogaster*. In *D. suzukii* and *D. melanogaster* all types of substrate vibrations recorded in intact males, were emitted also by males with amputated wings. We discuss the possibility that vibrational signalling may be widespread among *Drosophila* species. 

## Materials and Methods

### Insect rearing

Wild *D. suzukii* and *D. melanogaster* populations were collected in Northern Italy (San Michele all’Adige, Trento Province). No specific permits were required for collecting *D. suzukii* and *D. melanogaster*, since they are not endangered or protected species. Sampling locations were not privately owned or environmentally protected. A population of *D. biarmipes* was established from stock (14023-0361.09, San Diego Drosophila species Stock Centre). The three species were reared on a semiartificial diet prepared from yeast, flour, sugar and water, at a temperature of 23-25°C, relative humidity of 65±5 and 18:6 hr. L:D photoperiod. In order to obtain virgin individuals, newly emerged flies were removed several times per day from the tubes with the larval diet. In 15 *D. suzukii* and *D. melanogaster* males, the wings of newly emerged flies were surgically removed with microscissors under a stereo microscope. Care was taken that the wing was completely removed. In all behavioural experiments 3-7 day old individuals were used.

### Signal recording and analysis

Pairs of flies (one male and one female) for *D. melanogaster* and *D. biarmipes* or trios (one male and two females to increase the otherwise low courtship activity) for *D. suzukii* were placed into a recording arena, consisting of a round plastic frame (diameter 2 cm; height 0.5 cm) covered on top and bottom with fine netting, which allowed observation of fly behaviour. A piece of reflective tape was attached to the netting, to provide a surface on which to focus the beam of a laser vibrometer (Ometron VQ-500-D-V, Harpenden, UK). Recorded vibrations were digitized with a 48 kHz sample rate and 16- bit resolution, and stored directly onto a computer hard drive using LAN-XI data acquisition hardware (Brüel and Kjær Sound & Vibration A/S, Nærum, Denmark). Behaviour was simultaneously recorded with camcorder (Panasonic HDC-TM700, Hamburg, Germany) equipped with macro lens (Raynox dcr-25) in order to associate behaviour and body movements with emission of vibrational signals. Behaviour and substrate vibrations were recorded for 10 minutes or until copulation, whichever came first.

To describe substrate vibrations generated by each species, we determined the following parameters where appropriate: fundamental frequency (Hz), vibration intensity measured directly as substrate velocity (mm/s) using the Pulse 14 software (Brüel and Kjær Sound & Vibration A/S), signal duration (ms), and interpulse interval (IPI, time between two consecutive pulses). Spectral analysis of recorded vibrations was performed with Pulse 14. Recorded vibrations were analyzed with a Fast Fourier Transform (FFT) window length of 200 points and 90 % of overlap. To minimize the effect of the substrate on measured parameters, only those vibrations emitted by individuals within a range of 1 cm of the reflective tape were used for analyses.

To evaluate the differences in the parameters of the recorded substrate vibrations among species and the importance of the wings in the generation of substrate vibrations, the two-tailed t-test for unpaired data (Bonferroni corrected for multiple tests) [44] was used to compare vibrations emitted by the three species, and by winged and wingless *D. melanogaster* and *D. suzukii* males. 

## Results

**Figure 1:**
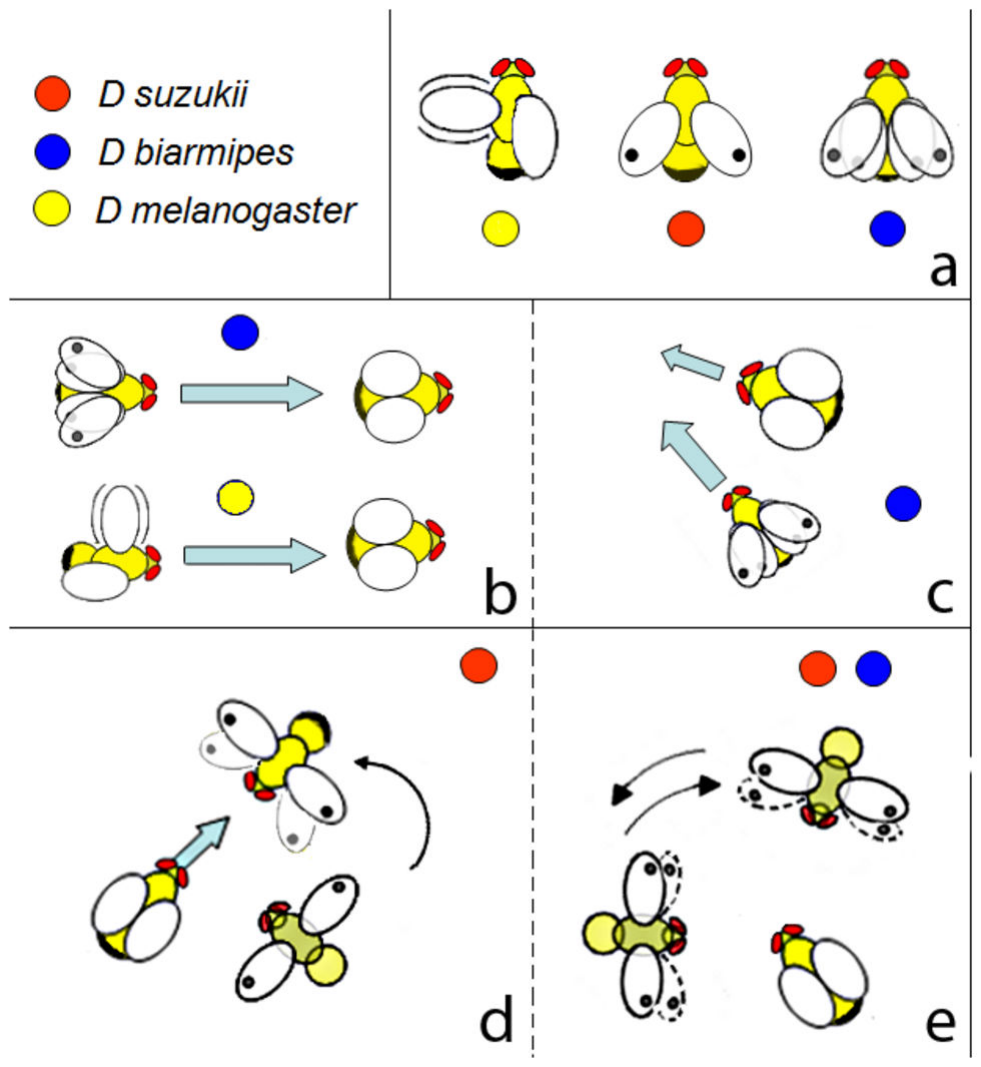
Behaviours accompanying emission of substrate vibrations in the three *Drosophila* species (*D. suzukii*, *D. biarmipes* and *D. melanogaster*). Coloured dots (red, blue and yellow) indicate different species. During chasing, males and females alternate phases of running with temporary stops during which (a) in *D. melanogaster* males vibrate one wing and emit pulse and sine songs, in *D. suzukii* males stand with wings semi-open, whereas in *D. biarmipes* males flutter their wings. During the phase of running, males may follow the females either from the back (*D. melanogaster*, *D. biarmipes*) (b), along the side (*D. biarmipes*) (c) or circle 45-90° around (*D. biarmipes*) or intercept (*D. suzukii*) them while extending either one or both wings (d, e). Toot emission in *D. suzukii* was always accompanied by wing extension and fast forward wing vibration (d), whereas in *D. biarmipes* it was accompanied by wing fluttering (a, b, c). For detailed information about vibrational signals emitted during these behavioural stages see Table 1.

Altogether, we recorded five distinct types of substrate vibrations generated by males during courtship, and their characteristics are reported in Tables 1 and 2. The features of some of the recorded substrate vibrations corresponded to previously recorded air-borne sounds. The repertoire differed among species. In *D. suzukii*, we recorded two types, in *D. melanogaster* three and in *D. biarmipes* four different substrate-borne sounds. In most cases, different substrate vibrations were associated with different behaviour (Table 1), and species differed in the expression of basic behaviours which were also accompanied by characteristic wing movements (Figure 1). Walking flies also induced incidental vibrations (Table 2). 

**Table 1:** Substrate vibrations recorded from the three *Drosophila* species. Numbers and letters refer to behaviour described in Figure 1.

	Abdominal quivering*	Wing ticking	Sine song	Pulse song	Toot
*D. melanogaster*	**a** **1	**-**	**a**, **b** ***2	**a**, b ***3	**-**
*D. suzukii*	a, d ****	**-**	**-**	**-**	d, e ****
*D. biarmipes*	a **1	e ****	e ****	**-**	**a**, **b**, c ***4

* Occurring also in “standing behaviour”.

** Previously described as substrate-borne signal ^1^:Fabre et al. 2012

*** Previously described only as air-borne signal ^2,3^: von Schilcher 1976 ^4^;Lai et al. 2009

Undescribed

**Table 2:** Temporal and spectral properties of substrate vibrations emitted by males during courtship, in *Drosophila melanogaster*, *D. suzukii* and *D. biarmipes*.

Type of vibration	Species	**N/n**	**Intensity (µm/s)**	**IPI (ms)**	**Duration (ms)**	**FF (Hz)**
Abdominal quivering	*D. melanogaster **wd***	9/10	4.7 ± 1.9 **a**	145 ± 19 b	-	-
	*D. melanogaster **wl***	9/10	5.0 ± 1.0	150 ± 17	-	-
	*D. suzukii **wd***	9/10	5.6 ± 1.6 **a**	77 ± 34 **a**	-	-
	*D. suzukii **wl***	9/10	5.8 ± 1.3	77 ± 41	-	-
	*D. biarmipes*	9/10	1.8 ± 1.0 b	69 ± 25 **a**	-	-
Pulse song	*D. melanogaster **wd***	10/10	34.1 ± 28.5	29 ± 2.0	-	-
	*D. melanogaster **wl***	10/10	29.8 ± 30.8	27 ± 1.5	-	-
Wing ticking	*D. biarmipes **wd***	5/12	21.0 ± 9.6	100 ± 17		-
“Toot”	*D. suzukii **wd***	10/2	3.8 ± 1.7 **a**	-	173 ± 38 **a**	302 ± 17 **a**
	*D. suzukii **wl***	10/2	3.9 ± 3.1	-	166 ± 33	304 ± 10
	*D. biarmipes*	10/2	82.7 ± 53.9 b	-	146 ± 32 **a**	450 ± 53 b
Sine song	*D. melanogaster **wd***	10/2	8.5 ± 8.7 **a**	-	1206 ± 552 b	191 ± 29 **a**
	*D. melanogaster **wl***	10/2	10.9 ± 21.8	-	1042 ± 424	206 ± 19
	*D. biarmipes*	10/2	3.8 ± 0.8 **a**	-	713 ± 400 **a**	199 ± 15 **a**
Incidental	*D. melanogaster*	5/3	12.0 ± 5.4	-	-	-
Vibrations	*D. suzukii*	5/3	12.6 ± 7.2	-	-	-
	*D. biarmipes*	5/3	13.3 ± 9.3	-	-	-

Means with standard deviation are shown.

N, number of animals; n, number of signals analysed for each individual; IPI = interpulse interval; FF = fundamental frequency; wd = intact males; wl = males with amputated wings. Different letters within each column indicate significant difference (p< 0.05) in a parameter for each song type between species (two tail t-test for unpaired data, with Bonferroni correction in the case of multiple comparison among the three species).

We divided male courtship behaviour (associated with emission of substrate vibrations in all three studied *Drosophila* species) into two main categories, chasing and standing. During the courtship sequence, periods of standing and chasing behaviour was observed to alternate, on occasion. 

**Figure 2:**
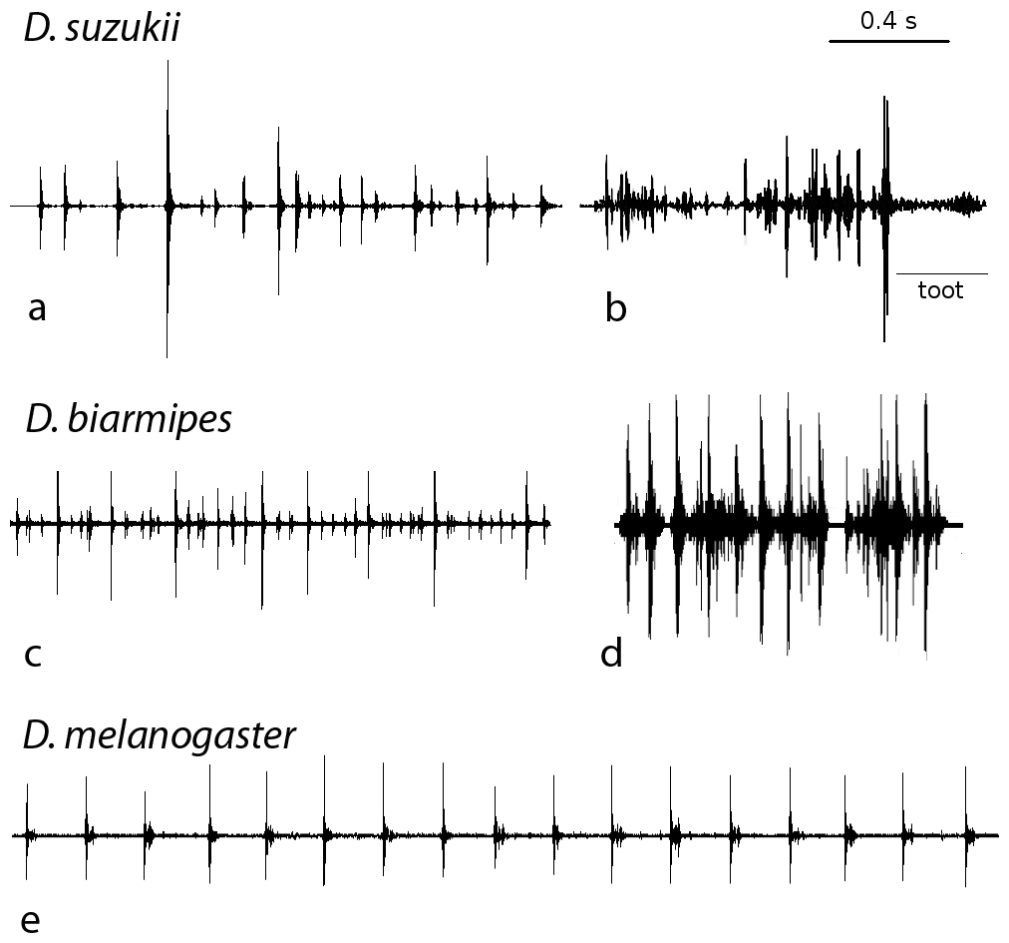
Oscillograms of abdominal quivering and wing ticking in three *Drosophila* species. a, b: abdominal quivering in *D. suzukii*. b shows accelerated quivering before emission of a "toot". c: abdominal quivering in *D. biarmipes*. d: wing ticking in *D. biarmipes*. e: abdominal quivering in *D. melanogaster*.

Behaviour was categorized as "standing" when both partners were immobile, and the male oriented with his head towards the female. Abdominal quivering [30] (Videos S1, S2, S7) was recorded in all three species (Figure 2), and was the only type of substrate vibrations recorded during standing for periods of variable duration, sometimes up to several minutes (maximum time observed for a single bout of standing: *D. melanogaster* 47 s; *D. suzukii* 112 s; *D. biarmipes* 376 s). Abdominal quivering was always associated with distinct dorso-ventral abdominal oscillations. In *D. melanogaster*, IPI in abdominal quivering was significantly longer and more regular than in the other two species (Table 2). Furthermore, in *D. suzukii* and *D. biarmipes*, pulse intensity was highly variable (Figure 2).

**Figure 3:**
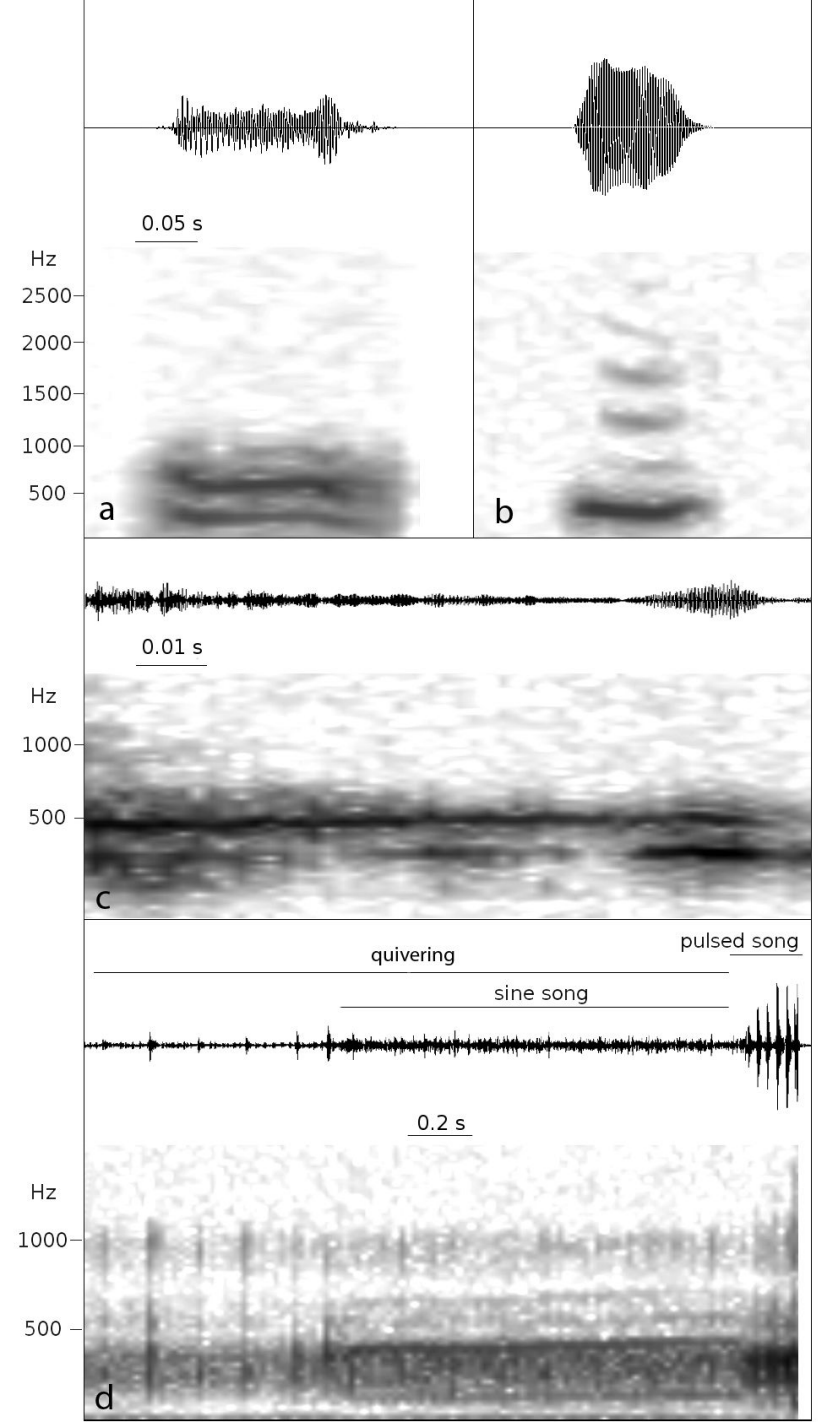
Oscillograms (above) and spectrograms (below) of vibrational components of (a) "toot" in *D. suzukii*, (b) "toot" in *D. biarmipes*, (c) sine song in *D. biarmipes*, (d) a sequence recorded in *D. melanogaster* which includes abdominal quivering, sine and pulse song.

Behaviour was categorized as chasing when male and female were moving, alternating with short periods (up to a few seconds) when females were immobile. During chasing, substrate vibrations were emitted by the male during the following basic behaviours: short periods of immobility (Figure 1a), following behind the female (Figure 1b), running on her side (Figure 1c) or running laterally either to intercept moving females frontally or to circle stationary females (Figure 1d, e). In chasing behaviour, *D. suzukii* males keep their wings partially or entirely opened, while *D. biarmipes* males flutter their wings; *D. melanogaster* males emit their songs during the female temporary stop (see below) (Figure 1a). Abdominal quivering was also observed during the short periods of immobility during chasing in all species, and in the case of *D. suzukii*, immediately before the emission of a so-called “toot” (see below) (Table 1; Video S3). In *D. melanogaster*, quivering often continued during the emission of a sine song (Figure 3d).

Other types of substrate vibrations were recorded during chasing. In *D. melanogaster*, the substrate component of the pulse song had the same temporal characteristics as had previously been determined for the air-borne component of this song [6,45]. The IPI was shorter than in abdominal quivering, and pulses had higher intensity (Table 2). The emission was often associated with vibration in one wing and a forward movement of the abdominal tip, similar to a mounting attempt. Although during the chase the substrate component of the pulse song was recorded more often than that of the sine song, these two types of substrate vibration were usually emitted in close association. Both types were recorded during moving, as well as during short periods of immobility. We recorded the substrate component of pulse and sine song when the male was following behind the female that was changing direction (Figure 1b). Pulse song emission was always accompanied by wing spreading in close proximity to the female (i.e. if the female turned left, the male opened the left wing). In contrast, the substrate component of sine song was recorded also in the absence of wing spreading. The vibrational component of a sine song was also found in *D. biarmipes* [37] (Figure 3c; Video S5) where it was emitted only during brief periods of female immobility during male circling. In this species, sine song emission was accompanied by extension of one or, more commonly, both wings, and by sideways body shaking sometimes associated with wing waving (Figure 1e). Both the duration and the fundamental frequency of the vibrational component of sine song in *D. biarmipes* differed significantly from the frequency and duration recorded in *D. melanogaster* (Table 2).

In *D. suzukii*, the rate of pulses in abdominal quivering sometimes accelerated just before the emission of a "toot" (Figure 2b). A "toot" is a sound signal characterized by frequency modulated sound with clear harmonic structure [36,37] (Figure 3a, b; Table 2). This type of substrate-borne sound was found in both species of the *suzukii* subgroup (Table 1, 2). Besides frequency structure, the “toot” in *D. suzukii* and *D. biarmipes* differed primarily in intensity, and in the behaviour associated with its emission. In *D. suzukii*, the "toot" was always emitted while the male was circling in close proximity to a temporarily stationary female, and it was accompanied by extension of one or both wings and a fast forward-directed wing vibration (Figure 1d; Videos S3, S4). In contrast, in *D. biarmipes*, the vibrational component of a “toot” was recorded when a male was either facing an immobile female or running behind (or beside) the female (Figure 1a, b, c; Video S7), and was always associated with fast wing fluttering.

A distinct type of substrate vibration described as “wing ticking” was found only in *D. biarmipes*, where it was recorded when males were circling around females (Figure 1e). It consisted of a short train of pulses associated with several forth and back thrusts of one wing (often alternating between right and left wing, but never with both together), and a movement of the abdominal tip to a downward position (Videos S5, S6). Compared to abdominal quivering, the IPI in wing ticking was longer and the pulse intensity was higher (Figure 2d; Table 2). 

In *D. suzukii* and *D. melanogaster*, all types of substrate vibrations found in intact males, were also recorded in experiments using males with amputated wings. No significant differences were found in most measured parameters in substrate-borne components of songs emitted by intact males and wingless males (Table 2). The sole exception was a slightly, but significantly, lower IPI in the substrate component of the wingless *D. melanogaster* pulse song (p < 0.05, two tail t-test for unpaired data).

## Discussion

In the present study, we demonstrate that substrate vibrations that differ markedly among species are associated with courtship behaviour in all three investigated *Drosophila* species. Besides Waldron [26], who suggested that females of *D. persimilis* perceive male song as substrate vibrations, other authors rarely mention vibrational signals emitted during *Drosophila* courtship. In *D. silvestris*, pulse song is not produced by wing vibrations, but by low-amplitude abdominal vibrations ("abdominal purring") [23]. In this species, wingless males did not show a reduced mating success [25], however, when substrate did not enable the transmission of vibrations, the courtship of this species was disrupted [27]. Potential vibrational signals produced by whole body vibrations (“trembles”) were also found in *D. tropicalis* and *D. eqinoxialis* [19]. In *D. melanogaster*, abdomen drumming has been mentioned, and although analysis showed that this behavioural component is an important element of a successful courtship, no further attention has been paid to this fact [28]. However, it has been shown recently that in *D. melanogaster*, *D. yakuba*, *D. sechellia*, as well as in *D. mauritania, D. simulans, D. biarmipes, D. mojavensis and D. willistoni* vibrational signals associated with abdominal quivering may trigger female receptivity and immobility [30]. It has also been suggested that in *D. melanogaster* incidental substrate-borne signals generated by a walking female may initiate the courtship [29].

Abdomen drumming in *D. melanogaster* was defined as "quickly repeated vertical movement of the abdomen which is tapped to the substrate" [28]. Vibrational signals generated by abdominal quivering [30] recorded in *D. melanogaster* most probably correspond to the abdomen drumming. Although the abdomen does not strike the substrate during production of vibrational signals described as abdominal quivering, it is easy to mistake the quick dorso-ventral movements for drumming, especially if flies are not viewed from the side. Production of vibrational signals by body vibrations transmitted to the substrate via the legs, has been described in many insects [21], including two dipteran families, Chloropidae and Agromyzidae [39,40]. Since substrate vibrations emitted by body vibrations have been found in different *Drosophila* subgroups (*melanogaster*, *willistoni*, *planitibia*, *suzukii, mulleri*) [19, 23, 30, the present study], it is conceivable that at least abdominal quivering may be present in most *Drosophila* species. 

Wing muscles are likely involved in the generation of the vibrational components of *Drosophila* air-borne sounds, such as pulse and sine songs, and “toots”. Thoracic vibrations accompanied with minute wing shivering have been associated with production of vibrational signals in the dipteran genera *Agromyza* and *Liriomyza* [40]. Muscle vibrations can either be transmitted to the substrate directly via the legs, or indirectly, as air-borne sound created by wing movement may induce substrate vibrations. Substrate-borne signals produced by wing fanning have been described in Hymenoptera [46]. As vibrational components of sine and pulse song and “toot” were recorded in experiments using males with amputated wings, the facts that the intensity of recorded vibrations did not differ between intact and wingless males, and that males of *D. biarmipes* do not spread wings when emitting a “toot”, suggest that direct transmission of vibrations via the legs is more likely than indirect transmission, via air. 

The main difference between *D. biarmipes* and *D. suzukii* observed in the present study was that in the latter, a "toot" was always accompanied with wing exposure, and therefore it seems that in this species, visual and acoustic cues are strictly combined. Of the two species, *D. suzukii* had an acoustic repertoire of only two , while *D. biarmipes* males of four different types of substrate-borne sounds. Of measured parameters only fundamental frequency of "toot" differed significantly between these two species. However, the IPI of abdominal quivering of both species in the *suzukii* subgroup differed significantly from *D. melanogaster*. It is also interesting to note that the IPI of abdominal quivering also seems to be less specific when compared within the *melanogaster* subgroup [30]. The less complex repertoire in *D. suzukii* in comparison with its sister species *D. biarmipes* [37] may either represent a loss as a result of an increased dependence on visual stimuli, differences in ecology of these two species, or a preserved ancestral state. It is currently believed that the common ancestor within the *D. melanogaster* group had spotted wings and that wing spots had been secondarily lost in the *D. melanogaster* subgroup [47]. 

"Toot" sounds seem to be characteristic of flies in the *suzukii* subgroup, since this type of sound has also been described in *D. pulchrella* [36,37]. Our results indicate that the fundamental frequency of the toot may be species-specific. The fundamental frequency of the *D. biarmipes* toots measured in our study as substrate-borne sound did not differ from the fundamental frequency of the air-borne component measured previously [36]. Furthermore, it seems that the change of frequency during the toot may also be species-specific, since, in contrast to the other two studied species, in *D. pulchrella* frequency is continuously falling throughout the "toot" [36]. The function of the "toot" in courtship has not been properly investigated, however, it has been noted that in *D. biarmipes* it may serve to attract the attention of the female [37]. Songs can evolve rapidly within the *Drosophila* species complex [19] and as has previously been shown in other insects, structurally similar vibrational songs can have different function in different species [48]. 

Our recordings also showed that incidental vibrations induced by moving flies may provide continuous background information throughout the courtship. Although incidental vibrations produced by locomotion may mask other vibrational signals used in intraspecific communication, some beetles use them to localize the females [49]. In this respect, it seems worthwhile to note that the much louder vibrational component of a "toot" was often produced by *D. biarmipes* males when both partners were running, while the quieter "toot" in *D. suzukii* is emitted when females are immobile, potentially to avoid interference from background noise. 

With a velocity threshold between 10^-5^ to 10^-6^ m/s, the subgenual organ (SGO) is the most sensitive insect organ that detects substrate vibrations [50,51], and so far, it has been found in all pterygote insects except in Coleoptera and Diptera [21]. However, *Drosophila* flies possess other, less sensitive mechanoreceptors that respond to vibrations, like numerous campaniform sensilla (CS) on their legs [27], as well as the femoral chordotonal organ (FCO) [52]. The lowest threshold values for the CS and FCO determined in other insects were between 10^-4^ and 10^-5^ m/s [51,53]. In the present study, all recorded values for substrate vibrations generated by *Drosophila* flies were above the threshold values of SGO; however, only the intensity of recorded vibrational components of pulse song in *D. melanogaster*, the “toot” and wing ticking in *D. biarmipes*, as well as incidental vibrations in all three species were above the threshold values of FCO and CS. There is currently no information about the physiological properties of vibration receptors in *Drosophila*, but there is behavioural evidence that females of *D. melanogaster* likely perceive abdominal quivering [30]. Furthermore, since attenuation of vibrational signals is highly dependent on the substrate [54,55], it is possible that the intensity recorded on an artificial substrate like netting may underestimate the intensity achieved on a natural substrate. 

Courtship behaviour in *Drosophila* is complex, and involves different sensory modalities. Available evidence suggests that substrate vibrations should be included among them. Given the importance of vibrational signalling in insect communication, further work may reveal *Drosophila* as an ideal model to study various aspects of vibrational communication. *Drosophila* species are ecologically highly divergent [2,33], and the relative importance of vibrational channel during courtship may reflect the use of a specific host (i.e. substrate). In the present study, we did not investigate the effect of recorded substrate vibrations generated by males on female behaviour. The function and relative importance of substrate vibrations in *Drosophila* courtship can be established only by playback experiments in which the role of visual, chemical and air-borne sound signals should be excluded. In this respect, it should also be emphasized that such experiments should be done on natural substrates to assure biologically relevant conditions. Furthermore, *D. suzukii* is recently receiving lot of attention due to its highly destructive pest status, as it is the only *Drosophila* species that lays eggs on fresh undamaged fruits, facilitated by its serrated ovipositor [32,33,56]. This ecological innovation, and the close relationship of *D. suzukii* with one of the most studied model species, *D. melanogaster*, provide excellent opportunities for addressing some longstanding questions in the field of insect biology, related to application in pest control. Furthermore, mating disruption based on substrate-borne signals may provide an environmentally safe strategy for *D. suzukii* management [57].

## Supporting Information

Video S1 **A Drosophila melanogaster pair.** While the female is grooming, the male with amputated wings performs a long abdominal quivering, with emission of both pulse and sine songs.
(AVI)

Video S2 **A Drosophila *suzukii* pair.** Male abdominal quivering during the courtship.
(AVI)

Video S3 **A Drosophila *suzukii* pair.** A male emits a series of "toots" while facing a female.
(AVI)

Video S4 **A Drosophila *suzukii* pair.** Typical male courtship behaviour: a male runs sideways towards a female with both wing wide open, occasionally emitting "toots" and abdominal quivering.
(AVI)

Video S5 **A Drosophila *biarmipes* pair.** Typical male courtship behaviour: a male faces a stationary female with wings wide open and then emits wing ticking followed by sine song.
(AVI)

Video S6 **A Drosophila *biarmipes* pair.** Detail of male wing ticking.
(AVI)

Video S7 **A Drosophila *biarmipes* pair.** Male abdominal quivering, followed by "toot" emission.
(AVI)
